# Association of granulocyte macrophage colony-stimulating factor and interleukin-17 levels with obsessive–compulsive disorder: a case–control study findings

**DOI:** 10.1038/s41598-023-46401-8

**Published:** 2023-11-03

**Authors:** Rapty Sarker, M. M. A. Shalahuddin Qusar, Sardar Mohammad Ashraful Islam, Mohiuddin Ahmed Bhuiyan, Md. Rabiul Islam

**Affiliations:** 1 Department of Pharmacy, University of Asia Pacific, 74/A Green Road, Farmgate, Dhaka, 1205 Bangladesh; 2 Department of Psychiatry, Bangabandhu Sheikh Mujib Medical University, Shahabagh, Dhaka, 1000 Bangladesh; 3 School of Pharmacy, BRAC University, 66 Mohakhali, Dhaka, 1212 Bangladesh

**Keywords:** Biochemistry, Neuroscience, Medical research

## Abstract

Obsessive–compulsive disorder (OCD) is a mental condition that affects many people and is characterized by recurring obsessions and compulsions. It significantly impacts individuals’ ability to function ordinarily daily, affecting people of all ages. This study aimed to investigate whether or not the cytokines granulocyte macrophage colony-stimulating factor (GM-CSF) and interleukin-17 (IL-17) are involved in the pathophysiology of OCD. A case–control study with 50 OCD patients and 38 healthy volunteers served as the controls for this investigation. The levels of GM-CSF and IL-17 in the serum of both groups were measured with enzyme-linked immunosorbent assay (ELISA) kits. In addition, the sociodemographic characteristics of the population under study were studied. Based on the findings of this study, OCD patients had significantly elevated levels of IL-17 than the controls, it appears that there may be a function for IL-17 in the pathophysiology of OCD. It was also discovered that the severity of OCD and IL-17 levels had a significant positive correlation. On the other hand, when comparing the levels of GM-CSF, there was no significant difference between the patients and the controls. This study provides evidence supporting the involvement of cytokine IL-17 in the pathophysiology of OCD. This study suggests IL-17 as a diagnostic biomarker for OCD and adds to our knowledge of the function that the immune system plays in this condition.

## Introduction

Obsessive–compulsive disorder (OCD) is a chronic mental health disorder characterized by recurring obsessions (intrusive, recurring, and distressing ideas or images frequently related to concerns about cleanliness, order, violence, or a constant need for acceptance) and compulsions (repetitive behaviours or mental acts for reducing worry or prevent harm, such as compulsive cleaning, inspecting, counting, or organizing objects) ^1,2^. Individuals with OCD struggle to control or ignore these obsessions and find relief through engaging in compulsive behaviours. It typically begins in childhood, adolescence, or early adulthood, and its symptoms can fluctuate in intensity over time ^3^. OCD is estimated to affect 2% of the world’s population and affects individuals of various ages, genders, and socioeconomic backgrounds. The World Health Organization (WHO) defined OCD as disability-adjusted life years (DALYs) in people between the ages of 15 to 44 and listed it as one of the top 20 disorders. According to the International OCD Foundation, 1 in 200 children and 1 in 40 adults suffer from OCD ^4–6^. This prevalence makes OCD one of the most common mental health disorders, surpassing conditions such as schizophrenia, bipolar disorder, and panic disorder. OCD can also become chronic if untreated, causing long-term disability and burdening healthcare systems. OCD also has a significant financial burden because it frequently necessitates medical treatment, counselling, and medication, all of which have high healthcare expenses ^7^. OCD can also affect work performance and diminish production, increasing economic burden ^8^. The primary treatments for OCD include cognitive-behavioural therapy (CBT), and medication, such as selective serotonin reuptake inhibitors (SSRIs), which can help alleviate symptoms by rebalancing neurotransmitter levels in the brain ^9,10^.

The pathogenesis of OCD involves complex interactions between genetic, neurobiological, and environmental factors ^11^. Although the exact mechanisms are unknown, numerous vital components have been identified. It is considered that a critical role is played by dysfunction in the cortico-striato-thalamo-cortical (CSTC) circuit, which involves communication between the prefrontal cortex, basal ganglia, and thalamus ^12–14^. Neurotransmitters such as serotonin, dopamine, and glutamate are implicated in OCD pathogenesis ^9^. Genetic influences are also present, with some gene polymorphisms increasing OCD risk. Environmental factors, such as traumatic experiences as a child or stressful living circumstances, may influence the onset or amplification of OCD symptoms ^15,16^. Furthermore, immune system dysregulation and inflammation, as evidenced by altered cytokine levels, have been implicated in the pathogenesis of OCD ^17^. It has been discovered that cytokines, immune system signalling molecules, contribute to the bidirectional communication between the immune and central nervous systems (CNS) ^18,19^. Research indicates that cytokine dysregulation, particularly of pro-inflammatory cytokines, may play a role in the pathophysiology of several mental diseases such as major depressive disorder, schizophrenia, bipolar disorder, anxiety disorders, and post-traumatic stress disorder, including OCD ^20–25^. Cytokine levels can trigger neuroinflammation, interfere with neurotransmitter functions, reduce neuroplasticity, and influence symptoms’ emergence and persistence ^26^.

Granulocyte–macrophage colony-stimulating factor (GM-CSF) is a cytokine that plays a critical role in regulating and differentiating immune cells, particularly granulocytes and macrophages ^27^. While there has not been much research on the exact connection between GM-CSF and OCD, increasing evidence suggests its potential role in the pathophysiology of OCD. According to studies, GM-CSF levels are different in people with OCD compared to healthy controls. GM-CSF levels are higher in OCD patients in several studies, pointing to a pro-inflammatory scenario ^28,29^. The increase in inflammation and the modulation of immunological responses have been linked to GM-CSF. It can stimulate and activate immune cells, which may affect the immune system dysregulation and neuroinflammation observed in OCD. Additionally, GM-CSF has been connected to modifications in synaptic activity and neuroplasticity, both of which are relevant to the OCD pathophysiology ^30,31^. Again, IL-17, a proinflammatory cytokine, has been implicated in autoimmune and inflammatory conditions and plays a crucial role in immune responses. It promotes inflammation and the recruitment of immune cells by synthesizing more cytokines and chemokines ^32,33^. Additionally, it has been proposed that IL-17 affects neuroimmune interactions and blood–brain barrier permeability, which may impact immune and CNS communication ^34^. Inflammatory bowel illness, rheumatoid arthritis, and psoriasis have all been linked to abnormal IL-17 production ^35,36^. Recent studies point to the potential role of IL-17 in OCD. According to studies, people with OCD had higher blood levels of IL-17 than healthy controls ^37,38^. The exact mechanisms by which IL-17 contributes to OCD are still being investigated.

OCD currently lacks specific assessment techniques, unlike many other conditions with clinical assessment methods available for diagnosis and monitoring ^39^. For this reason, OCD diagnosis heavily relies on clinical assessment, which can be subjective and prone to variability. However, the need for establishing reliable diagnostic methods, such as biomarkers, to assist in diagnosing and monitoring OCD is crucial. The establishment of objective biomarkers, such as markers for neuroimaging or particular genetic markers, may improve diagnostic precision and facilitate early action ^40,41^. These biomarkers may help distinguish OCD from other psychiatric diseases with comparable symptoms and provide insights into the neurobiological mechanisms in action. Biomarkers may also help monitor therapy effectiveness and predict the condition’s long-term course, enabling specific treatment plans for OCD patients. To find the precise causes of OCD and reliable biomarkers, however, there is still considerable work to be done despite research advances. To increase diagnostic accuracy, improve strategies for treatment, and ultimately provide better care for OCD patients, it is essential to research and identify biomarkers for OCD.

Numerous studies have been done over the past few years to find potential OCD diagnostic indicators. This study aims to demonstrate a solid correlation between particular markers and the existence of OCD, which will eventually result in the development of quantitative diagnostic techniques. Since it is still unclear how cytokine changes relate to the pathophysiology of OCD. As a result, the current study used a case–control design to assess serum GM-CSF and IL-17 in OCD.

## Materials and methods

### Study population

This prospective case–control study included 50 OCD patients and 38 healthy controls. The patients were chosen from the psychiatry department of the Bangabandhu Sheikh Mujib Medical University (BSMMU) hospital in Dhaka, Bangladesh; the controls were chosen from different areas throughout the city. To establish a comparable study population, the controls and patients were carefully matched based on age, gender, and socio-demographic variables. The DSM-5 criteria were used to make the OCD diagnosis. Using the Yale-Brown obsessive–compulsive scale (Y-BOCS), the severity of OCD symptoms was evaluated. A standardized questionnaire was used to collect both groups’ socio-demographic characteristics and clinical evaluations. People between the ages of 18 and 60 with a Y-BOCS score of eight or higher met the inclusion criteria for the study. People having a Y-BOCS score of less than eight, cognitive impairment, acute or significant medical diseases, a history of liver or kidney failure, and associated psychiatric disorders were all excluded from the study. Individuals with chronic infectious diseases or a recent history of infection preceding the past 30 days were also excluded from the study. These strict requirements were used to guarantee a homogeneous study population and to reduce confounding variables that could affect the study results.

### Sample collection

A total of 5 ml of blood was drawn from the cephalic vein of each participant. After collection, the blood was allowed to clot in Falcon tubes. Then the tubes were centrifuged at 3000 rpm for 15 min to obtain the serum. To guarantee optimal storage and preservation, the acquired serum was carefully collected in Eppendorf tubes and kept in a refrigerator at a temperature of − 80 °C to preserve the samples’ integrity.

### Measurement of serum GM-CSF and IL-17

Using commercially available enzyme-linked immunosorbent assay (ELISA) kits purchased from BosterBio, USA, the levels of GM-CSF and IL-17 in the blood were measured. These ELISA kits were selected because of their precision and specificity in detecting the desired cytokines. The same investigators performed the entire experiment procedure to preserve consistency and reduce potential biases. Notably, the experiments were carried out by researchers unaware of the outcomes, guaranteeing objective data analysis.

### Yale-Brown obsessive–compulsive scale

The Y-BOCS is a widely used clinical tool for measuring the severity of OCD. It involves obsessions and compulsions, which are the two main domains and are both rated from 0 to 4. Obsessions are evaluated in terms of the time spent on these intrusive thoughts, their interference with daily activities, the distress they cause, attempts to resist them, and the level of control over them. Compulsions are similarly assessed based on time spent on these repetitive behaviors, interference with daily life, anxiety when prevented from performing them, attempts to resist compulsions, and control over these behaviors. The total score divides OCD severity into four categories: mild (scoring 8–15), moderate (scoring 16–23), severe (scoring 24–31), and extreme (scoring 32–40). It is an essential tool that supports clinical practice and research in diagnosing and monitoring OCD ^42,43^.

### Statistical analysis

The Statistical Package for Social Sciences (SPSS) software, version 25.0, and Microsoft Excel were used to process the data and perform statistical analysis. To compare the various groups and evaluate the relationships between variables, independent samples t-tests, Chi-square tests, and correlation tests were used. Boxplot graphs were made to illustrate the data graphically. Socio-demographic profiles were calculated using descriptive analysis, and the results were shown as mean ± SEM (standard error mean). Receiver operating characteristic (ROC) curve analysis was also performed to evaluate the diagnostic performance analysed parameters. A p-value of 0.05 or below was considered statistically significant, denoting a significant difference between groups or a significant correlation between variables.

### Ethical considerations

The study protocol was approved by the research ethics committee of University of Asia Pacific (UAP/REC/2023/105). Informed consent was obtained from all the study participants. We performed this study following the Helsinki Declaration.

## Results

### Characteristics of study population

The characteristics of study participants are presented in Table 1. In terms of age distribution, the majority of both patients and controls fell into the 18–25 and 26–35 age ranges (patients: 40.00%, controls: 42.11%; p = 0.924) in each category. There were slightly more males than females in both groups (patients: 66.00% males, controls: 63.16% males; p = 0.825). Regarding marital status, the majority of patients and controls were unmarried (patients: 58.00%, controls: 68.42%; p = 0.428). In terms of education level, the highest proportion in both groups was graduates and above (patients: 44.00%, controls: 47.37%; p = 0.772). Most participants in both patients and controls were unemployed (patients: 26.00%, controls: 31.58%; p = 0.498). The economic impression was mainly medium for both groups (patients: 72.00%, controls: 89.47%; p = 0.281). The majority of patients and controls were from urban areas (patients: 56.00%, controls: 68.42%; p = 0.348). Most participants were non-smokers (patients: 86.00%, controls: 94.74%; p = 0.311). The most common BMI category for both groups was 18.5–25.0 (patients: 70.00%, controls: 57.89%; p = 0.327). In this study, the presence of previous history and family history of OCD in patients was 50.00% and 34.00%, respectively. On the other hand, there was no such presence of previous history and family history of OCD in healthy controls.

**Table 1:** Characteristics of the study population.

Parameters	OCD patients n (%)	Healthy controls n (%)	p value
Sex			0.825
Male	33 (66.00)	24 (63.16)	
Female	17 (34.00)	14 (36.84)	
Age in years			0.942
18–25	20 (40.00)	16 (42.11)	
26–35	20 (40.00)	16 (42.11)	
36–45	5 (10.00)	2 (5.26)	
46–60	5 (10.00)	4 (10.53)	
Marital status			0.428
Married	21 (42.00)	12 (31.58)	
Unmarried	29 (58.00)	26 (68.42)	
Education level			0.772
Illiterate	2 (4.00)	2 (5.26)	
Primary	7 (14.00)	6 (15.79)	
Secondary	5 (10.00)	0 (0.00)	
Higher secondary	14 (28.00)	12 (31.58)	
Graduate and above	22 (44.00)	22 (47.37)	
Occupation			0.498
Business	10 (20.00)	2 (5.26)	
Housewife	10 (20.00)	8 (21.05)	
Student	7 (14.00)	10 (26.32)	
Unemployed	13 (26.00)	12 (31.58)	
Others	10 (20.00)	6 (15.79)	
Economic status			0.281
High	2 (4.00)	0 (0.00)	
Medium	36 (72.00)	34 (89.47)	
Low	12 (24.00)	4 (10.53)	
Area of residence			0.348
Rural	22 (44.00)	12 (31.58)	
Urban	28 (56.00)	26 (68.42)	
Smoking history			0.311
Non-smoker	43 (86.00)	36 (94.74)	
Smoker	7 (14.00)	2 (5.26)	
BMI (kg/m^2^)			0.327
Below 18.5 (CED)	2 (4.00)	0 (0.00)	
18.5–25.0 (Normal)	35 (70.00)	22 (57.89)	
Above 25.0 (Obese)	13 (26.00)	16 (42.11)	
Previous history of OCD			< 0.001
Yes	25 (50.00)	0 (0.00)	
No	25 (50.00)	38 (100.00)	
Family history of OCD			< 0.001
Yes	17 (34.00)	0 (0.00)	
No	33 (66.00)	38 (100.00)	

*OCD* obsessive–compulsive disorder, *BMI* body mass index, *CED* chronic energy deficiency.

### Clinical profile and laboratory findings

In the clinical profile and laboratory findings (Table 2), the mean age of the patients was 30.10 ± 1.46 years, while the controls had a mean age of 29.37 ± 2.53 years (p = 0.797). The mean BMI for patients was 23.72 ± 0.72 kg/m^2^, and for controls, it was 23.95 ± 0.8 kg/m^2^ (p = 0.858). The patients exhibited significantly higher Y-BOCS scores compared to the healthy controls (p ≤ 0.001). The serum levels of GM-CSF in the patients were lower (282.97 ± 34.28 pg/ml) compared to the healthy controls (325.53 ± 89.28 pg/ml), although no statistical significance was found (p = 0.587).

**Table 2:** Clinical features and laboratory findings of the study participants.

Parameters	OCD patients (n = 50) Mean ± SEM	Healthy controls (n = 38) Mean ± SEM	p value
Age (in years)	30.10 ± 1.46	29.37 ± 2.53	0.797
BMI (kg/m^2^)	23.72 ± 0.72	23.95 ± 0.8	0.858
Y-BOCS score	25.70 ± 1.01	–	< 0.001
Serum GM-CSF levels (pg/ml)	282.97 ± 34.28	325.53 ± 89.28	0.587
Serum IL-17 levels (pg/ml)	108.49 ± 14.45	57.31 ± 4.23	0.034

*BMI* body mass index, *DSM-5* diagnostic and statistical manual for mental disorders, 5th edition, *Y-BOCS* Yale-Brown obsessive–compulsive disorder scale, *GM-CSF* granulocyte macrophage colony stimulating factor, *IL-17* interleukin 17, *OCD* obsessive–compulsive disorder, *SEM* standard error mean.

**Figure 1:**
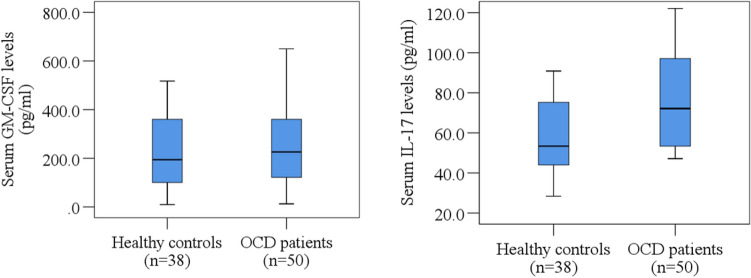
Distribution of serum GM-CSF and IL-17 levels in OCD patients and healthy controls. Boxplot graphs showing the median, maximum, and minimum value range.

**Figure 2:**
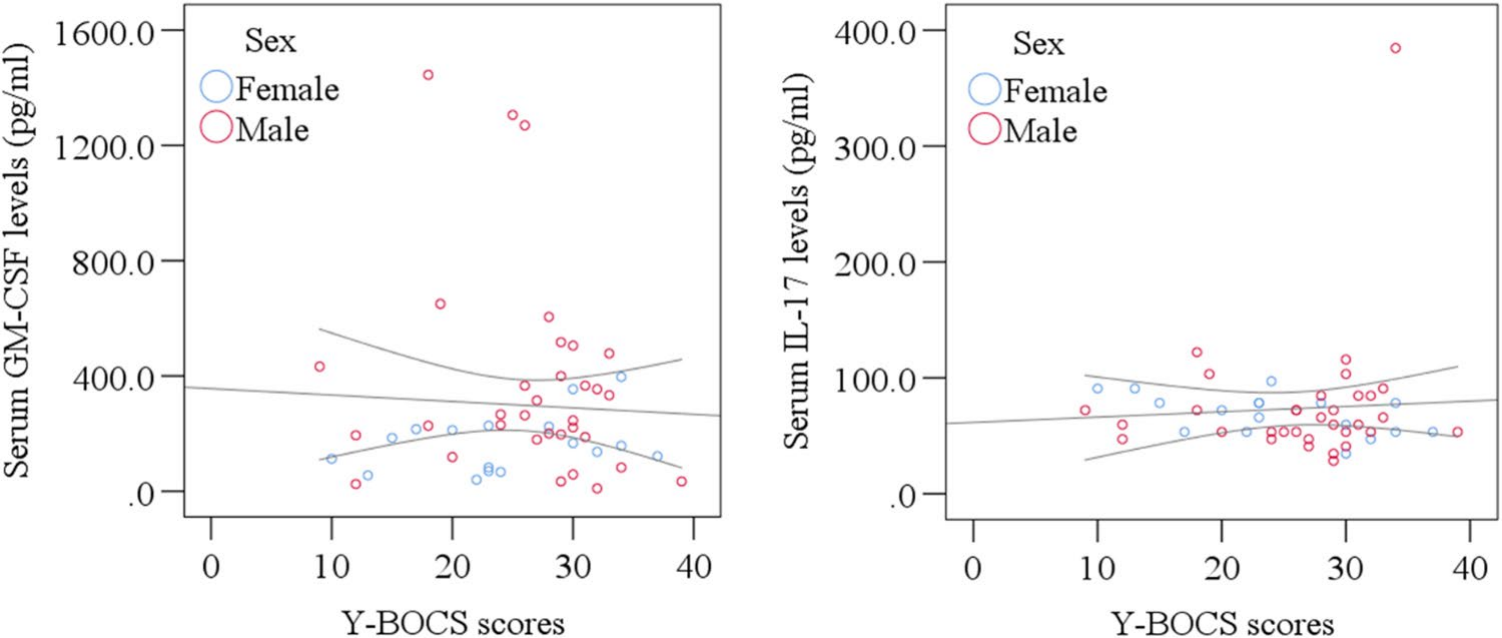
Sex-specific scatter plot graphs showing the association and mean difference of serum GM-CSF and IL-17 levels with Y-BOCS scores of OCD patients.

On the other hand, the patients had higher serum IL-17 levels (108.49 ± 14.45 pg/ml) compared to the healthy controls (57.31 ± 4.23 pg/ml), which was found statistically significant (p = 0.034). The distribution of serum GM-CSF and IL-17 levels in OCD patients and healthy controls has been presented in Fig. 1. Furthermore, there was a significant correlation between OCD severity and serum IL-17 levels (p = 0.037). Sex-specific scatter plot graphs also presented the association and mean difference of serum GM-CSF and IL-17 levels with Y-BOCS scores of OCD patients in Fig. 2.

### Correlation analysis

We performed Spearman’s correlation analysis among various research parameters in OCD patients (Table 3). Also, we performed Bonferroni corrections for all p-values for this pairwise comparison. We observed a significant positive association between serum IL-17 levels and Y-BOCS scores of OCD patients (r = 0.361; p = 0.012). However, no significant positive or negative associations were observed for other parameters (p > 0.05).

**Table 3:** Spearman’s correlation study among various research parameters among OCD patients.

Correlation parameters	*r*	p*
Age and Y-BOCS score	0.127	0.380
Age and GM-CSF	0.233	0.103
Age and IL-17	0.221	0.123
BMI and Y-BOCS score	− 0.182	0.207
BMI and GM-CSF	0.146	0.311
BMI and IL-17	0.043	0.768
GM-CSF and Y-BOCS score	− 0.263	0.162
IL-17 and Y-BOCS score	0.361	0.012
GM-CSF and IL-17	0.137	0.345

*BMI* body mass index, *GM-CSF* granulocyte macrophage colony-stimulating factor, *IL-17* interleukin-17, *OCD* obsessive–compulsive disorder, *Y-BOCS* Yale-Brown obsessive–compulsive scale.

*Bonferroni-corrected p values.

### Receiver operating characteristic curve analysis

**Figure 3:**
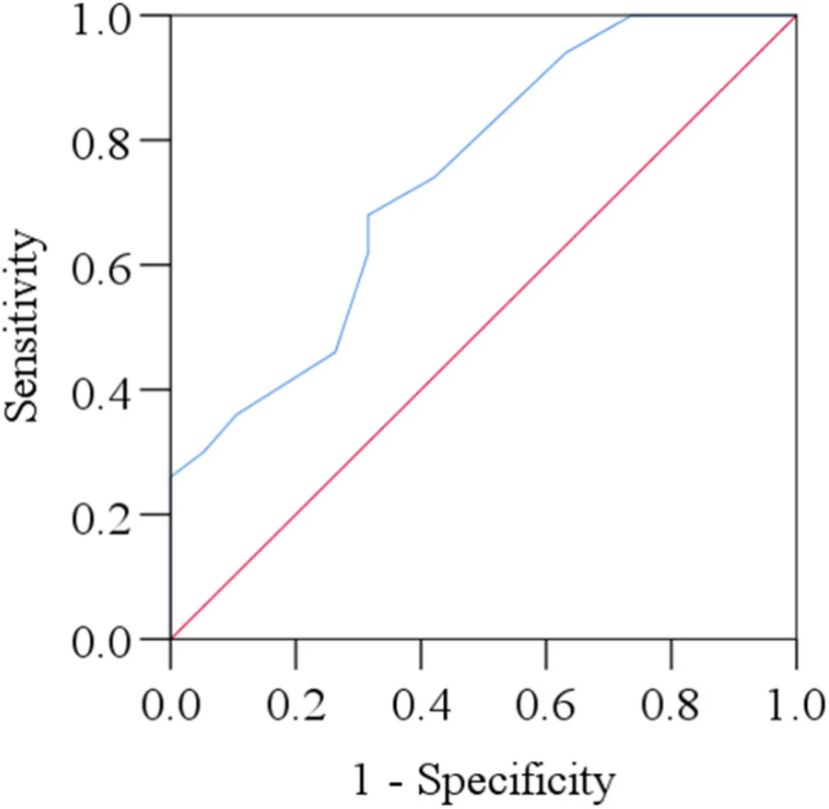
Receiver operating characteristic (ROC) curves of serum IL-17 levels among the study population.

The results of ROC curve analysis of serum IL-17 levels have been presented in Table 4 and Fig. 3. According to the analysis, the cut-off value was 62.74 pg/ml. Sensitivity and specificity were observed at 68.0% and 68.4%. The area under the curve (AUC) was reported at 0.742 with a p-value of 0.002.

**Table 4:** Receiver operating characteristic curve analysis of serum IL-17 levels.

Parameters	Cut-off value (pg/ml)	Sensitivity (%)	Specificity (%)	AUC	95% CI		p-value
					Lower bound	Upper bound	
IL-17	62.74	68.0	68.4	0.742	0.610	0.874	0.002

*AUC* area under the curve, *CI* confidence interval, *IL-17* interleukin-17.

## Discussion

This study analysed the sociodemographic profiles and biophysical characteristics of the study population to understand the factors associated with OCD. The findings showed no significant differences between the patients and controls regarding age, sex distribution, marital status, BMI, education level, occupation, economic impression, region of residence, or smoking history. This shows that these factors may not directly influence the development or manifestation of OCD and that the groups were evenly distributed concerning these characteristics, reducing their possible impact on the study’s findings.

Regarding the laboratory findings, there was no significant difference between the patients and controls regarding serum GM-CSF levels. This shows that GM-CSF may not significantly impact the pathophysiology of OCD in the population under study. In contrast, Rodriguez et al. found a higher level of GM-CSF level in OCD patients than in healthy controls ^28^. On the other hand, the analysis of serum IL-17 levels indicated a significant difference between the patients and controls, with higher levels observed in the patients’ group. Again, when examining the relationship between OCD severity and GM-CSF levels within the OCD patient group, no significant correlation was found. While our findings showed a significant positive correlation between the levels of IL-17 and OCD severity, in contrast to GM-CSF. Levels of IL-17 increased with increasing OCD severity. This data raises the possibility that IL-17 has a role in the pathophysiology of OCD. The observed increase in IL-17 levels with worsening OCD symptoms may be due to an immunological and inflammatory imbalance unique to OCD.

This data suggests that IL-17 plays a role in the immunological dysregulation and inflammatory processes connected to OCD. As IL-17 is a proinflammatory cytokine, it can influence neurotransmitter systems implicated in OCD, such as serotonin, glutamate, and dopamine. Serotonin, in particular, is a crucial neurotransmitter in regulating mood and behaviour. Proinflammatory cytokines can interfere with serotonin synthesis, release, and reuptake, resulting in imbalances that intensify OCD symptoms. These cytokines can also affect glutamate and dopamine signalling, which has an additional impact on the pathophysiology of OCD ^44–47^. Furthermore, neuroinflammation caused by these cytokines can disrupt brain circuits involved in cognitive flexibility and emotional processing, which would help develop and maintain OCD symptoms ^48,49^. Immune dysregulation and autoimmune processes are other possible routes connecting cytokines to OCD ^18^. According to research, some people with OCD have more autoantibodies targeting particular brain proteins, such as anti-streptococcal antibodies. These antibodies can elicit an immunological response, which then produces cytokines and causes neuroinflammation ^50^. The resulting neuroimmune interactions may contribute to the development and complications of OCD symptoms.

As there are currently no reliable quantitative diagnostic techniques or identified biomarkers for OCD. Nonetheless, IL-17 could function as an effective assessment tool for the evaluation of OCD as of the present study. By measuring IL-17 levels in individuals with suspected or confirmed OCD, clinicians can gain valuable insight into the immunological dysregulation and inflammatory processes related to the disorder. This biomarker may help with OCD diagnosis, tracking the development of the condition, and evaluating treatment effectiveness. Clinical reasoning can be improved by using IL-17 as an assessment tool, enabling more individualized and targeted therapy methods for OCD patients. Additionally, the discovery of IL-17 can help in understanding the pathophysiology of OCD by illuminating the function of immune dysregulation and inflammation in the onset and maintenance of the condition. Overall, using IL-17 as a biomarker in OCD can increase diagnostic precision, better therapeutic approaches, and deepen our comprehension of the disease’s underlying mechanisms.

This study also conducted ROC analysis (Fig. 3) which showed a moderate sensitivity and specificity levels indicating the diagnostic accuracy of biomarker in distinguishing OCD patients from non-OCD individuals. The AUC, indicating diagnostic utility, was in the fair range ^22^, while a statistically significant p-value underscored the significance of the observed AUC. The implications of this ROC analysis for the diagnosis and therapy of OCD are promising. Incorporating serum IL-17 levels as a diagnostic biomarker could enhance diagnostic accuracy, facilitating earlier detection and intervention. The identification of a specific cut-off value enables the stratification of patients into risk groups, aiding in the development of personalized treatment plans. Early intervention based on IL-17 levels may lead to better outcomes by reducing OCD severity and progression ^51,52^. Additionally, considering complementary biomarkers or clinical parameters alongside IL-17 could improve overall diagnostic accuracy, offering a more comprehensive diagnostic profile for OCD. While IL-17 is not extensively studied in the context of OCD, some research has explored its potential involvement in the disorder. Similar to this study, some studies also found a higher level of IL-17 in OCD. However, while their study showcases compelling insights, it regrettably falls short in terms of serving as a viable risk assessment tool, primarily due to its lack of predictive performance capabilities ^37,38^.

This study’s findings suggest several potential therapeutic approaches for individuals with OCD. Immunomodulatory treatments targeting IL-17 could regulate immune response and reduce inflammation. Serotonin-targeted therapies, including SSRIs, may be beneficial given IL-17’s impact on serotonin synthesis. Glutamate and dopamine modulators could address neurochemical imbalances associated with OCD. Integrating IL-17 knowledge into CBT may tailor strategies for immune-related factors. IL-17 as a biomarker allows personalized treatment plans for better outcomes. Early intervention based on IL-17 could facilitate timely treatment and improve the prognosis. Adopting a balanced diet, regular exercise, stress reduction techniques, adequate sleep, limiting stimulants, avoiding substance abuse, building a support network, and practicing mindful awareness can aid in managing OCD symptoms.

### Limitations

A few limitations should be acknowledged in this study. Firstly, the sample size was limited, and participants with a smoking habit were not excluded. This could introduce confounding factors that may interfere with cytokine analyses, potentially influencing the results. Furthermore, this study did not consider essential factors such as food habits, physical activity, and sleep patterns. To enhance the comprehensiveness of this study, a broader range of both anti-inflammatory and pro-inflammatory interleukins should have been investigated. Therefore, it is crucial to interpret our findings cautiously, as they should be considered preliminary. To establish a more robust understanding, further studies with a larger and more homogeneous population, accounting for confounding variables specific to depression, and a broader range of cytokines are recommended in this field.

## Conclusion

This study aimed to evaluate the possible function that two cytokines, GM-CSF and IL-17, could play as biomarkers for OCD. The findings contribute to the ongoing efforts that are being made to establish practical diagnostic tools for this medical condition and provide valuable insights into the pathophysiology of OCD. The detection of accurate biomarkers might increase the accuracy of OCD diagnosis, make it easier to take a tailored approach to treatment, and lead to better patient results. Further research is required to elucidate the underlying mechanisms connecting cytokine dysregulation, neuroinflammation, and OCD, which will pave the way for novel therapeutic interventions and a better understanding of this complex disorder.

## Data Availability

The data supporting the present study findings are obtainable from corresponding authors upon reasonable request.
